# Myricetin Attenuates Hyperexcitability of Trigeminal Nociceptive Second-Order Neurons in Inflammatory Hyperalgesia: Celecoxib-like Effects

**DOI:** 10.3390/molecules30183789

**Published:** 2025-09-18

**Authors:** Sana Yamaguchi, Mamoru Takeda

**Affiliations:** Laboratory of Food and Physiological Sciences, Department of Life and Food Sciences, School of Life and Environmental Sciences, Azabu University, 1-17-71, Fuchinobe, Chuo-ku, Sagamihara 252-5201, Kanagawa, Japan; f22027@azabu-u.ac.jp

**Keywords:** inflammation, trigeminal nociceptive neuron, hyperalgesia, pathological pain, extracellular single-unit recording, celecoxib, NSAIDs, flavonoids, myricetin, complementary alternative medicine

## Abstract

Myricetin (MYR), a naturally occurring flavonoid widely distributed in fruits and vegetables, was investigated for its potential to reduce inflammation-induced hyperexcitability in the spinal trigeminal nucleus caudalis (SpVc), which is associated with hyperalgesia. The study also compared MYR’s impact with that of celecoxib (CEL), a non-steroidal anti-inflammatory drug (NSAID). To induce inflammation, Complete Freund’s adjuvant was injected into the whisker pads of rats. Subsequently, we measured the mechanical escape threshold by applying mechanical stimuli to the orofacial region. We found that inflamed rats exhibited a significantly lower threshold compared to naive rats (each group, n = 4). This reduced threshold returned to the naive level two days after the administration of MYR (16 mg/kg, i.p.), CEL (10 mg/kg, i.p.), and a combination of MYR (8 mg/kg, i.p.) + CEL (5 mg/kg, i.p.). To investigate the nociceptive neural response to orofacial mechanical stimulation, we performed extracellular single-unit recordings to measure the activity of SpVc wide-dynamic range (WDR) neurons in anesthetized subjects. In inflamed rats, administration of MYR, CEL, or 1/2MYR + 1/2CEL (each group, n = 4) significantly reduced both the average spontaneous activity and the evoked firing rate of SpVc neurons in response to non-painful and painful mechanical stimuli. The increased average receptive field size in inflamed rats was normalized to the naive level following treatment with MYR, CEL, or 1/2MYR + 1/2CEL. These findings suggest that MYR administration can mitigate inflammatory hyperalgesia by reducing the heightened excitability of SpVc WDR neurons. This supports the notion that MYR could be a viable therapeutic option in complementary and alternative medicine for preventing trigeminal inflammatory mechanical hyperalgesia, potentially serving as an alternative to selective cyclooxygenase-2 blockers.

## 1. Introduction

Small Aδ-fibers and unmyelinated C-fibers from trigeminal ganglion neurons transmit painful sensory signals from the orofacial region to second-order neurons in the spinal trigeminal nucleus caudalis (SpVc) [1,2,3,4]. The functional classification of SpVc nociceptive neurons includes nociceptive-specific and wide-dynamic range (WDR) types according to their responses to mechanical stimuli in orofacial regions like the facial skin. Importantly, SpVc WDR neurons respond to both painful and non-painful stimuli [3,4]. The application of graded noxious stimuli to their receptive fields elicits an increased firing frequency in SpVc WDR neurons that is dependent on stimulus intensity, indicating their critical involvement in encoding stimulus intensity. Rat models of orofacial inflammation, induced by Complete Freund’s adjuvant (CFA), are a standard method for investigating trigeminal neural pathways related to pathological pain. In these models, we observe hyperexcitability of SpVc wide-dynamic range (WDR) neurons in response to mechanical stimuli [5,6]. These findings, along with previous reports linking SpVc neurons to dental pain and hyperalgesia [1,3,4,5], highlight the importance of the SpVc in orofacial pain mechanisms.

In Western countries, a growing number of patients unresponsive to conventional Western medical treatments are turning to complementary and alternative medicine (CAM), particularly for managing chronic pain [7]. Given these findings, CAMs show potential in preventing trigeminal inflammatory hyperalgesia. This aligns with prior research demonstrating that the hyperactivity of SpVc WDR neurons, which contributes to inflammation-associated pain sensitivity, can be dampened by the long-term intake of dietary components, including polyphenols and carotenoids. Through the inhibition of both peripheral and central cyclooxygenase-2 (COX-2) signaling pathways [8,9], natural products from fish, vegetables, and fruits demonstrate analgesic effects, including for inflammatory pain. Myricetin (MYR), a flavonoid polyphenol, is present in various fruits and vegetables. It is well-known for its numerous beneficial biological effects, including antioxidant, anti-inflammatory, and anticancer properties [10,11,12]. In vitro studies have shown that MYR can inhibit the secretion of glutamate, an excitatory neurotransmitter, from cerebral cortex neuron nerve terminals in a concentration-dependent manner. This effect is primarily achieved by suppressing voltage-gated calcium (Cav) channels [13].

Furthermore, in vitro studies show that MYR lowers the discharge frequency of hypothalamic paraventricular neurons by activating voltage-gated K^+^ (Kv) channels [14]. By acting on both presynaptic and postsynaptic membranes, MYR enhances the efficacy of the inhibitory neurotransmitter γ-aminobutyric acid (GABA) in central nervous system neuronal preparations [15]. Additionally, MYR has been shown to cross the blood–brain barrier [16]. We recently demonstrated that acute intravenous administration of MYR, in the absence of inflammatory or neuropathic pain, produces a short-term inhibition of SpVc WDR neuronal excitability, including nociception. The observed effects are likely mediated by the inhibition of Cav channels and activation of Kv channels. Thus, MYR is a promising therapeutic agent for trigeminal nociceptive pain, potentially offering a safer alternative to current treatments [17].

MYR inhibits the production of inflammatory cytokines such as prostaglandin E2 (PGE2), tumor necrosis factor-α, and interleukin-1β [18]. As these mediators are generated in inflamed tissues and amplify the inflammatory response, MYR’s ability to inhibit them is significant. Furthermore, MYR also suppresses the production of cyclooxygenase-2 (COX-2), the key enzyme for PGE2 synthesis [19,20]. PGE2 is known to induce pain hypersensitivity by sensitizing mechanosensitive channels (e.g., transient receptor potential ankyrin 1 (TRPA1) and acid-sensing ion channel (ASIC)) and voltage-dependent Na (Nav) and Kv channels. This occurs through protein kinase A (PKA) activation, mediated by prostanoid E (EP) receptors at nociceptive terminals [21], and by decreasing the activity of glycinergic inhibitory interneurons in the spinal dorsal horn [22]. Naringenin, a phytochemical found in citrus fruits, alleviates inflammatory hyperalgesia by reducing the hypersensitivity of nociceptive SpVc WDR neurons that encode pain intensity [9]. This effect results from the inhibition of the COX-2 signaling cascade, primarily at the site of inflammation. Furthermore, naringenin’s pain-relief effects have been confirmed to be comparable to non-specific COX-2 inhibitors like the non-steroidal anti-inflammatory drug (NSAID) diclofenac [9]. Diclofenac is a widely prescribed NSAID with proven analgesic, anti-inflammatory, and antipyretic properties, effectively treating various acute and chronic pain and inflammatory conditions [23]. Diclofenac inhibits both COX-1 and COX-2, whereas celecoxib (CEL) is a selective inhibitor of COX-2. To our knowledge, no reports currently link dietary chemical components with the specific COX-2 inhibitor, CEL [24]. Together, these observations strongly suggest that MYR administration attenuates inflammation-induced hyperexcitability of SpVc WDR neurons associated with trigeminal hyperalgesia, indicating its potential as a therapeutic agent for preventing inflammatory hyperalgesia. Therefore, the present study used behavioral and electrophysiological techniques to investigate whether MYR administration under in vivo conditions attenuates inflammation-induced hyperexcitability of SpVc neurons associated with hyperalgesia in rats. Additionally, we examined and compared the potency of MYR and NSAIDs, specifically the COX-2 inhibitor celecoxib (CEL), in suppressing hyperalgesia-associated, inflammation-induced SpVc neuronal excitability. We hypothesized that replacing a half-dose of conventional CEL with a half-dose of MYR could contribute to effective CAM therapies while reducing potential side effects. MYR administration was found to attenuate inflammatory hyperalgesia by reducing the hyperexcitability of SpVc WDR neurons. These findings suggest MYR’s potential as a therapeutic agent for preventing trigeminal inflammatory mechanical hyperalgesia within CAM strategies.

## 2. Results

### 2.1. Inflammation-Induced Hyperalgesia

**Figure 1:**
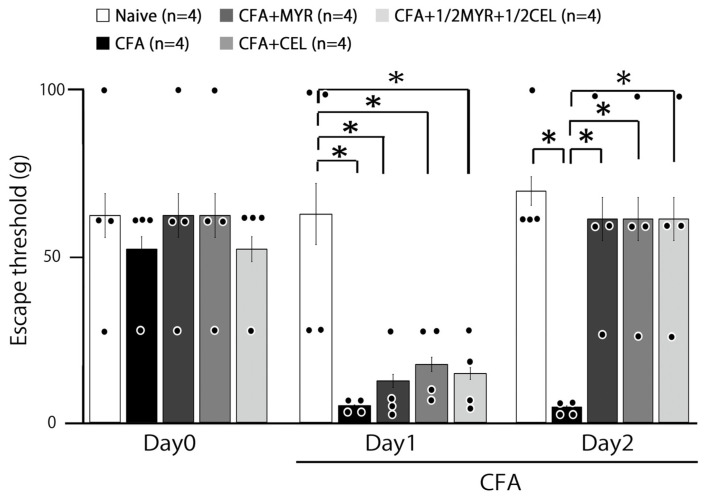
Effects of myricetin (MYR), celecoxib (CEL), and their combination on mechanical escape threshold in inflamed rats. Mechanical stimulation with von Frey hairs was applied to the ipsilateral whisker pad to assess hyperalgesia in five groups of rats: naive (saline, n = 4), Complete Freund’s adjuvant (CFA)-inflamed (n = 4), CFA-inflamed treated with MYR (16 mg/kg, i.p., n = 4), CFA-inflamed treated with CEL (10 mg/kg, i.p., n = 4), and CFA-inflamed treated with a half-dose combination of CEL and MYR (5 mg/kg, CEL + 8 mg/kg MYR, i.p., n = 4). Data represent changes in escape threshold over time. Statistical evaluation was performed using one-way repeated-measures analysis of variance (ANOVA), followed by Tukey–Kramer. Closed circle indicates the distribution of individual data points. *, *p* < 0.05.

Following CFA injection into the whisker pad, we assessed hyperalgesia in rats by probing the injection site and/or facial skin. As illustrated in Figure 1, the mechanical escape threshold was significantly reduced in the inflamed whisker pad region, from 70.0 ± 4.3 g in naive rats to 4.5 ± 0.4 g on Day 2 post-injection (n = 4, *p* < 0.05; Figure 1). Our findings are consistent with previous studies, which showed no significant changes in naive rats after vehicle injection [8,9]. Furthermore, no notable changes in the contralateral whisker pad threshold were found between the two groups (naive vs. inflamed; 63.2 ± 3.8 g vs. 56.9 ± 4.7 g, n = 4, NS).

### 2.2. Chronic Treatment with Myricetin, Celecoxib, and Their Combination for Hyperalgesia

Daily MYR administration partly restored the reduced mechanical escape threshold in inflamed rats on Day 1 (Figure 1). On Day 2, however, the CFA-induced decrease in the threshold was fully reversed by MYR administration, returning it to naive levels (70.0 ± 4.3 g vs. 66.5 ± 3.7 g, n = 4, NS; Figure 1). Similarly, CEL administration also fully restored the threshold to naive levels on Day 2 (70.0 ± 4.3 g vs. 61.5 ± 6.6 g, n = 4, NS). No significant difference was found between the MYR and CEL treatment groups. Furthermore, the co-administration of a half-dose combination (1/2 CEL + 1/2 MYR) was also effective, fully restoring the threshold to naive levels on Day 2 (naive vs. Day 2 inflamed + 1/2 CEL + 1/2 MYR; 70.0 ± 4.3 g vs. 63.0 ± 7.6 g, n = 4, NS). This indicates no significant difference between naive rats and inflamed rats administered with the combined treatment at Day 2.

### 2.3. Myricetin (MYR) Reduces Inflammatory Edema: Whisker Pad Thickness

**Figure 2:**
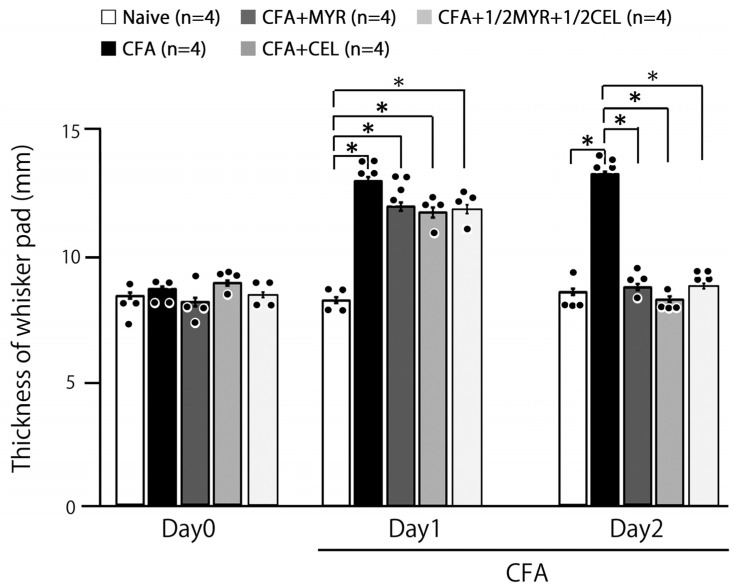
Effects of myricetin (MYR), celecoxib (CEL), and their combination on whisker pad thickness in inflamed rats. The thickness of the ipsilateral whisker pad was measured to assess the inflammatory response in five groups of rats: naive (saline; n = 4), Complete Freund’s adjuvant (CFA)-inflamed (n = 4), CFA-inflamed treated with MYR (16 mg/kg, i.p.; n = 4), CFA-inflamed treated with CEL (10 mg/kg, i.p.; n = 5), and CFA-inflamed treated with a half-dose combination of CEL and MYR (e.g., 5 mg/kg CEL + 8 mg/kg MYR, i.p.; n = 4). Data are presented as mean ± SEM. Statistical evaluation was performed using one-way repeated-measures analysis of variance (ANOVA), followed by Tukey–Kramer. Closed circle indicates the distribution of individual data points. *, *p* < 0.05.

CFA injection significantly increased whisker pad edema thickness in inflamed rats compared to naive controls, with the effect sustained from Day 1 to Day 2 (*p* < 0.05). On Day 1, the average thickness in inflamed rats was 13.0 ± 0.2 mm, compared to 8.3 ± 0.1 mm in naive rats (n = 4, Figure 2). Importantly, MYR, CEL, and 1/2 CEL + 1/2 MYR administration effectively reversed the CFA-induced edema, restoring whisker pad thickness to control levels by Day 2 (naive vs. inflamed + MYR: 8.5 ± 0.2 mm vs. 8.6 ± 0.3 mm, n = 4, NS; Figure 2).

### 2.4. Altered Excitability of SpVc WDR Neurons Following Inflammation

**Figure 3:**
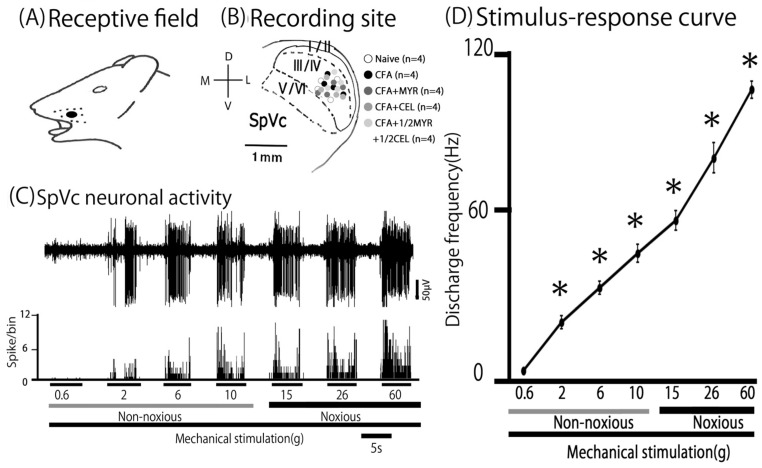
General characteristics of spinal trigeminal nucleus caudalis (SpVc) wide-dynamic range (WDR) neuronal activity in orofacial skin. (**A**) Representative receptive field of a whisker pad neuron in the facial skin. (**B**) Somatotopic distribution of SpVc WDR neurons (n = 20) responsive to non-noxious and noxious mechanical stimulation of the facial skin. Layers I–VI of SpVc. (**C**) Example traces illustrating non-noxious and noxious mechanical stimulation-induced firing of a SpVc WDR neuron. (**D**) Stimulus–response curve for SpVc WDR neurons (n = 20). Data represent mean firing frequency ± SEM. * *p* < 0.05 for comparison of 2 g stimulus versus 6 g, 10 g, 15 g, 26 g, and 60 g stimuli. Statistical evaluation was performed using Student’s *t*-test.

Mechanical stimulation of the whisker pad activated a total of 20 SpVc WDR neurons in rats from both the naive and inflamed groups, and the treatment groups (inflamed + MYR, inflamed + CEL, and 1/2 CEL + 1/2 MYR). As shown in Figure 3A, these neurons, which responded to both non-noxious and noxious mechanical stimuli, exhibited a somatic receptive field in the whisker pad. The neurons were distributed in layers III-V (n = 20) of the SpVc and were typically found in the maxillary branch (Figure 3B). No obvious differences in their distribution were observed among the recording sites of the three groups. As depicted in a stimulus–response graph (Figure 3D), the firing rate of the SpVc neuron increased upon application of varying intensities of mechanical stimulation to the most sensitive area within its receptive field (Figure 3C). Therefore, as described in our previous studies [8,9], every neuron analyzed was categorized as a WDR neuron (Figure 3C).

**Figure 4:**
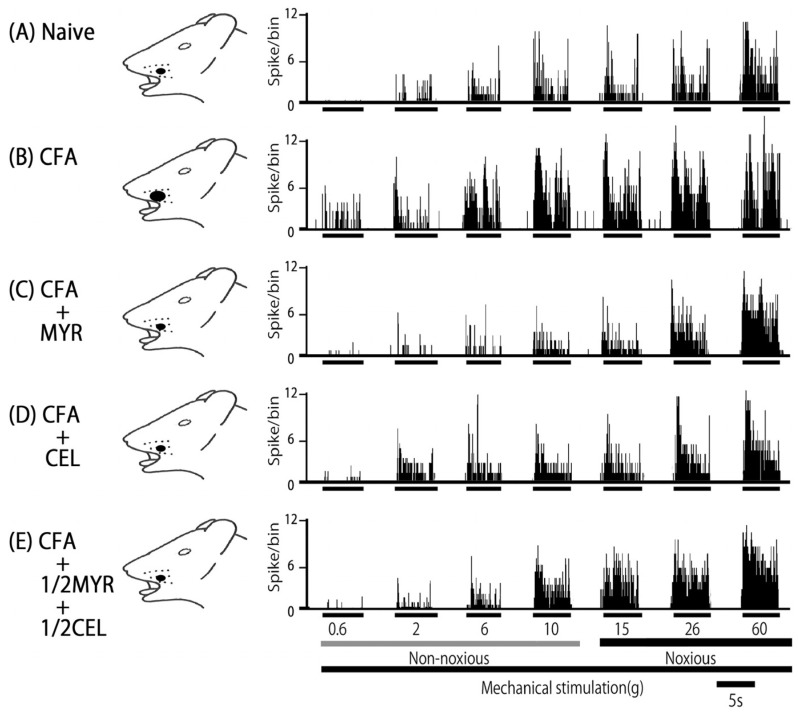
Chronic myricetin (MYR) and celecoxib (CEL) administration reverses hyperexcitability of spinal trigeminal nucleus caudalis (SpVc) wide-dynamic range (WDR) neurons following orofacial CFA inflammation. Representative traces show non-noxious and noxious mechanical stimulation-induced firing of SpVc WDR neurons from various rat groups: naive (n = 4), CFA-inflamed (n = 4), CFA-inflamed treated with MYR (16 mg/kg, i.p. for 2 days; n = 4), CEL (10 mg/kg, i.p. for 2 days; n = 4), or a half-dose combination of CEL and MYR (e.g., 5 mg/kg CEL + 8 mg/kg MYR, i.p. for 2 days; n = 4). Note that the decreased mechanical stimulation threshold, increased spontaneous discharges, and expanded receptive field size observed in inflamed rats were normalized to control levels following two days of MYR or CEL administration.

**Figure 5:**
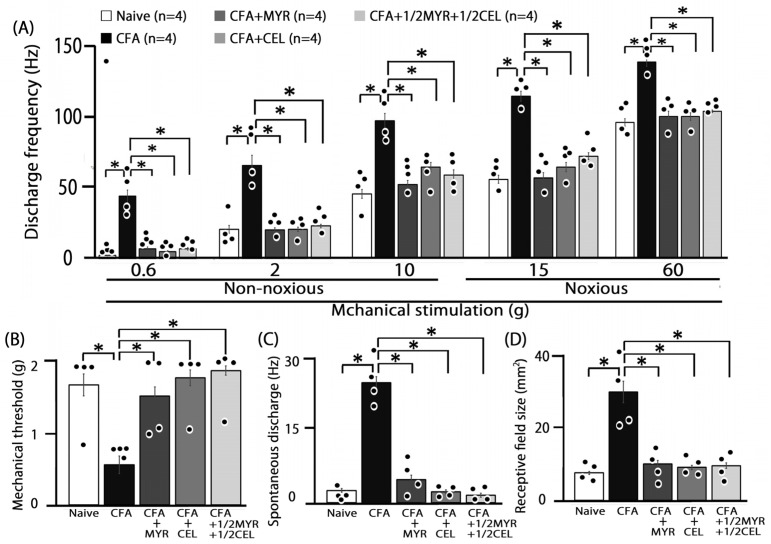
Chronic myricetin (MYR) and celecoxib (CEL) treatment reverses SpVc WDR neuronal hyperexcitability induced by orofacial CFA inflammation. This figure presents a summary of key electrophysiological parameters of SpVc WDR neurons (n = 4 rats per group) following chronic administration of MYR or CEL in orofacial CFA-inflamed animals. (**A**) Comparison of the mean evoked discharge frequency of SpVc WDR neurons in response to mechanical stimulation (non-noxious and noxious) of the orofacial skin. (**B**) Assessment of the mean mechanical threshold required to activate SpVc WDR neurons. (**C**) Quantification of the spontaneous discharge frequency of SpVc WDR neurons. (**D**) Evaluation of the mean receptive field size of SpVc WDR neurons. For all panels (**A**–**D**), statistical significance is indicated by an asterisk (*), representing *p* < 0.05 for pairwise comparisons: naive vs. inflamed rats and inflamed vs. inflamed rats treated with MYR, CEL, or the 1/2 CEL + 1/2 MYR combination. Statistical evaluation was performed using one-way repeated-measures analysis of variance (ANOVA), followed by Tukey–Kramer. Closed circle indicates the distribution of individual data points.

Consistent with our prior studies, we first confirmed that CFA induces hyperexcitability in SpVc WDR neurons [8,9]. In naive rats, spontaneous discharges were observed in 75% (3/4) of SpVc neurons (Figure 4 and Figure 5C), with most neurons exhibiting low-frequency firing, averaging 2.8 ± 0.3 Hz (n = X). Conversely, all WDR neurons in inflamed rats (4/4; 24.8 ± 1.3 Hz) were spontaneously active (Figure 4 and Figure 5C). Compared to naive rats, SpVc WDR neurons in inflamed rats displayed markedly enhanced responses to non-noxious mechanical stimuli (Figure 4B and Figure 5A), consistent with our previous findings [8,9]. The mean firing rates of SpVc WDR neurons in response to mechanical stimuli (0.4, 2, 15, 60 g) were notably higher in inflamed rats compared to control rats (n = 4; Figure 5A). Furthermore, the mean mechanical threshold in inflamed rats significantly decreased to 0.6 ± 0.1 g from 1.7 ± 0.2 g in naive rats (n = 4; Figure 5B). Inflammation-induced changes were not limited to evoked responses. Inflamed rats exhibited a significantly elevated spontaneous discharge frequency compared to naive rats (Figure 5C). This hyperexcitability was accompanied by a significant increase in the mean receptive field size, which grew from 7.5 ± 0.4 mm^2^ in naive rats to 29.8 ± 2.9 mm^2^ in inflamed rats (n = 4, *p* < 0.05; Figure 5D).

### 2.5. Chronic Myricetin (MYR) Inhibits SpVc WDR Neuronal Hyperexcitability in Inflamed Rats

To investigate whether prolonged MYR treatment affects the hyperexcitability of SpVc WDR neurons in inflamed rats, we performed a behavioral analysis for the escape threshold on Day 2. Figure 4 illustrates this effect, presenting representative examples of SpVc WDR neuron responses to both non-noxious (0.6–10 g) and noxious (15–60 g) mechanical stimulation after MYR administration. Following two days of daily MYR administration in inflamed rats, the discharge rate of SpVc WDR neurons in response to both non-noxious and noxious mechanical stimulation returned to control values (Figure 4). The previously observed reduced mechanical threshold and increased spontaneous, noxious, and non-noxious firing frequencies in inflamed rats reverted to the levels seen in control rats. Figure 5A illustrates that MYR treatment significantly decreased the average discharge frequency of SpVc WDR neurons in inflamed rats in response to both non-noxious and noxious mechanical stimuli (*p* < 0.05). MYR administration effectively reversed the effects of inflammation on SpVc wide-dynamic range (WDR) neurons. As shown in Figure 5, MYR significantly restored the reduced mean mechanical stimulation threshold in inflamed rats to control levels (Figure 5B). It also significantly reduced the spontaneous firing of these neurons (*p* < 0.05, Figure 5C) and diminished the mean receptive field size to control levels (Figure 5D). These findings are consistent with our previous studies [8,9]. We found that vehicle administration did not notably affect either the spontaneous or mechanical stimulation-induced hyperactivity.

### 2.6. Chronic Celecoxib (CEL) Inhibits SpVc WDR Neuronal Hyperexcitability in Inflamed Rats

We next investigated how chronic CEL administration affects the hyperexcitability of SpVc wide-dynamic range (WDR) neurons in inflamed rats on Day 2. The effects of CEL administration on SpVc WDR neuron firing rates in response to non-noxious and noxious mechanical stimulation are illustrated in the representative examples shown in Figure 4. After two days of daily CEL administration, the discharge frequency of these neurons to both non-noxious and noxious mechanical stimulation returned to control levels (Figure 4). Specifically, CEL treatment reversed the inflammation-induced changes in inflamed rats (Figure 5), including the decreased mechanical threshold (Figure 5B) and the increased spontaneous, noxious, and non-noxious firing rates (*p* < 0.05). The average mechanical stimulation threshold, in particular, returned to control levels after CEL treatment (Figure 5B). Furthermore, the spontaneous activity of SpVc WDR neurons in inflamed rats significantly reduced following CEL administration (Figure 5C, *p* < 0.05). The average receptive field size in inflamed rats was also significantly reduced to the levels observed in the control group (Figure 5D).

### 2.7. Chronic Half-Dose Myricetin (MYR) + Celecoxib (CEL) Inhibits SpVc WDR Neuronal Hyperexcitability in Inflamed Rats

Our study analyzed the influence of chronic co-administration of half-dose CEL and half-dose MYR on the hyperexcitability of SpVc WDR neurons induced by inflammation on Day 2. Figure 4 shows representative examples of firing rates in SpVc WDR neurons exposed to non-noxious (0.6–10 g) and noxious stimuli (15–60 g), after the 1/2 MYR + 1/2 CEL administration. Following this combined treatment, the reduced mechanical threshold and increased spontaneous, noxious, and non-noxious firing frequencies observed in inflamed rats returned to control levels. As shown in Figure 5A, the average discharge frequency of SpVc WDR neurons in inflamed rats significantly declined after the 1/2 MYR + 1/2 CEL administration (*p* < 0.05). The mean mechanical stimulation threshold in inflamed rats also significantly returned to normal levels after this treatment (Figure 5B). Furthermore, the spontaneous firing rate of SpVc WDR neurons in inflamed rats was significantly reduced following the 1/2 MYR + 1/2 CEL administration (Figure 5C, *p* < 0.05). Inflamed rats also exhibited a significant reduction in mean receptive field size, reaching control levels (Figure 5D).

## 3. Discussion

### 3.1. Myricetin (MYR) Attenuates Trigeminal Inflammatory Hyperalgesia

The objective of this study was to determine if the systemic administration of MYR can alleviate the hyperexcitability of SpVc neurons induced by inflammation and its associated mechanical hyperalgesia. In this study, we made the following key findings: (i) CFA-inflamed rats exhibited a significant increase in whisker pad thickness and a lower escape threshold to orofacially applied mechanical stimulation compared to naive rats, consistent with previous reports [8,9]. (ii) Systemic administration of MYR for two days restored the increased whisker pad thickness and reduced mechanical threshold to control levels in CFA-inflamed rats. (iii) Vehicle administration had no significant effect on the escape threshold in two-day CFA-inflamed rats. A previous study reported that in vitro administration of MYR inhibits peptidoglycan-induced COX-2 expression in cardiac myocytes [19]. Consistent with prior research, our current findings indicate that dietary components like carotenoids can effectively reduce mechanical hyperalgesia caused by CFA-induced inflammation in rats. This effect is thought to be mediated by a reduction in COX-2-immunoreactive cells within the whisker pad [8]. Taken together, these observations suggest that daily administration of MYR reduces inflammation-induced hyperalgesia in the rat whisker pad, likely via COX-2 signaling suppression, which subsequently inhibits PGE2 production through previously described mechanisms [8]. A limitation of this study is that we only tested a single dose of MYR and CEL. Future research should therefore focus on investigating the dose–response relationships and therapeutic windows to fully understand their potential. While previous studies have shown that myricetin can cross the blood–brain barrier [16], a more definitive demonstration of its central nervous system activity is warranted. To this end, we believe that additional pharmacokinetic studies are essential. These studies should include the precise quantification of myricetin not only in its crystalline state, but also to determine its concentration within the central nervous system itself.

### 3.2. Myricetin (MYR) Suppresses SpVc WDR Neuronal Hyperexcitability in Inflammation-Induced Hyperalgesia

The four main steps of nociceptive sensory signaling begin with the initial transduction of external stimuli at peripheral terminals, followed by the generation and axonal propagation of action potentials, and finally transmission to central terminals that form the first synapses in the sensory pathways of the central nervous system [25]. A previous study reported that systemic MYR administration significantly and reversibly inhibited nociceptive SpVc WDR neuronal activity in a dose-dependent manner [17]. However, to our knowledge, the precise mechanism by which MYR suppresses inflammation-induced hyperexcitability of these neurons has not been investigated in a systemic in vivo electrophysiological study. This remains a critical gap in our understanding.

Peripheral inflammation and/or nerve injury initiate a cascade of events. Inflammatory mediators like PGE2 bind to E-type prostanoid receptors, activating protein kinase A (PKA) and protein kinase C (PKC) in nociceptive peripheral terminals. This in turn leads to the phosphorylation of mechanosensitive ion channels and receptors, such as TRPA1 and ASIC3 [4,21]. The phosphorylation lowers the activation threshold of these channels, increasing the membrane excitability of peripheral terminals. A high frequency of action potentials is thus conducted to the presynaptic central terminals of the SpVc; the release of large amounts of glutamate augments excitatory postsynaptic potentials (EPSPs) by binding to upregulated post-synaptic receptors. This process causes a barrage of action potentials to be conducted to higher pain centers, ultimately establishing a state of heightened sensitivity known as peripheral sensitization [4,21].

Our study reveals that daily systemic MYR administration effectively restores the decreased mechanical stimulation threshold in inflamed rats to control levels. This behavioral finding is well-supported by our electrophysiological data, which show that chronic MYR treatment significantly returns both non-noxious and noxious mechanical stimuli-evoked mean discharge frequencies of SpVc wide-dynamic range (WDR) neurons to control levels. These findings suggest that systemic MYR administration can modulate inflammation-induced hypersensitivity of SpVc WDR neuronal activity, likely by suppressing peripheral sensitization. This notion is further supported by the observed reversal of increased whisker pad thickness and the reduction in the mechanical threshold to control levels in CFA-inflamed rats by Day 2 of systemic MYR administration in the present study.

The classification of CaV channels broadly divides them into two groups: low-voltage-activated (T-type) and high-voltage-activated (L, P/Q, N, and R types) [26]. Both N-type and T-type Cav channels significantly mediate the primary afferent signaling pathway. High-voltage-activated N-type calcium channels are primarily located in the presynaptic regions of the dorsal horn (laminae I and II) [26,27,28]. The propagation of action potentials along C and Aδ afferents of dorsal root ganglion neurons triggers the opening of these channels, leading to the release of nociceptive neurotransmitters like glutamate, substance P, and calcitonin-gene-related peptide onto spinal interneurons and projection neurons [28]. Previous in vitro studies support this mechanism, showing that MYR inhibits glutamate release from rat cortex nerve terminals in a dose-dependent manner by suppressing presynaptic Cav and MAPK signaling cascades [28]. This effect was specifically abolished by N-, P-, and Q-type Ca^2+^ channel blockers [13]. Based on these findings and our current results, we propose that systemic MYR administration suppresses trigeminal nociceptive neuronal excitability by a mechanism involving N-type Ca^2+^ channels, likely located at the presynaptic terminals of primary trigeminal ganglion neurons under inflammatory conditions.

Kv channels are crucial for various neuronal functions, including regulating resting membrane potential, shaping action potentials, mediating repolarization, and controlling neurotransmitter release. These roles are facilitated by distinct channels, such as the slow-inactivating sustained (IK) and fast-inactivating transient (IA) currents [29,30,31,32,33]. Previous studies have shown that a reduction in IA channel density, but not IK channel density, increases the excitability of small-diameter neurons in rats in vivo [33]. Our previous work showed that the local application of the polyphenol, chlorogenic acid, reduces the discharge frequency of SpVc WDR neurons through the activation of Kv channels [34]. Consistent with these findings, MYR has been shown in vitro to reduce discharge frequency by promoting Kv channels in hypothalamic paraventricular neurons [14]. This channel activation induces hyperpolarization of the resting membrane potential, thereby diminishing neuronal excitability. Consequently, various types of Kv channels have been proposed as therapeutic targets for pain management [33,35]. Taken together, these findings and our current results suggest that systemic administration of MYR suppresses the excitability of trigeminal nociceptive neurons under inflammatory conditions. This effect is likely mediated by the activation of Kv channels, potentially at the postsynaptic SpVc neurons.

The current study demonstrated that MYR counteracted the elevated mean spontaneous discharge frequency of SpVc WDR neurons caused by inflammation. This finding is particularly significant given that previous reports have linked ongoing activity in the SpVc to enduring headaches (spontaneous pain) [36]. The prolonged activation of SpVc WDR neurons has been shown to stem from peripheral signals, with evidence from recent studies showing a significant reduction in continuous activity following lidocaine administration into the trigeminal ganglia [37]. Our results, when combined with these findings, suggest that MYR reduces the heightened spontaneous discharge activity of SpVc WDR neurons through peripheral and/or trigeminal ganglion sensitization.

Our previous research indicated that a local GABAergic mechanism can modulate nociceptive transmission in SpVc neurons, thereby influencing their general mechanical receptive field properties [38]. Our study demonstrated that MYR treatment reversed the increased receptive field size observed in inflamed rats, returning it to baseline. While the precise mechanisms for this effect remain to be elucidated, it is plausible that this finding is linked to MYR’s modulatory role on the central nervous system. Previous in vitro studies have shown that MYR enhances the efficacy of the inhibitory synaptic transmitter, GABA, by acting on both presynaptic and postsynaptic membranes [15].

Based on these findings, we hypothesize that MYR may regulate local GABAergic 8 tonic control and suppress excitatory synaptic transmission within the nociceptive pathway. This action could serve as a central mechanism to mitigate pain signaling. Our previous work demonstrated that changes in mechanical receptive field size resulted from the local iontophoretic application of GABA_A_ receptor agonists and antagonists [38]. Therefore, it is plausible that MYR reduces receptive field sizes by modulating (activating) GABAergic inhibitory mechanisms in the local SpVc circuit. Further studies are warranted to substantiate this hypothesis.

To substantiate the hypothesis regarding the mechanism by which myricetin alleviates inflammatory hyperalgesia, the following future research is required: (i) protein quantification using methods such as Western blotting to demonstrate that myricetin reduces the expression of COX-2 and PGE2, which are upregulated during inflammation in this experimental model; (ii) a comparison with selective channel inhibitors to prove whether Ca and Kv channels are involved in the suppression of neuronal activity by myricetin; and (iii) proof of the facilitatory effect of the GABA_A_ receptor mechanism on the recovery of receptive field size by myricetin.

To the best of our knowledge, a report by Ekstrand et al. [39] found sex-related differences in how selected flavonoids and phenolic compounds modify porcine hepatic cytochrome P450 (CYP450)-dependent activity. Specifically, they showed that MYR noncompetitively inhibited CYP3A activity only in microsomes from male pigs, while female pigs’ CYP3A remained unaffected. A prior investigation observed that experimental pain perception varies during the menstrual cycle, with increased sensitivity to several pain modalities during the luteal phase compared to the follicular phase [40]. Although the major role of sex hormones in pain variability is widely recognized, the specific mechanisms responsible for this disparity are still not fully comprehended [41]. Consequently, our hypothesis was tested exclusively on male rats. Subsequent studies are necessary to determine whether these findings hold true for the opposite sex.

### 3.3. Functional Significance of Myricetin (MYR)’s Suppressive Effect on SpVc Neuronal Hyperexcitability in Hyperalgesia

Herbal medicines, acupuncture, and other complementary and alternative medicine (CAM) therapies are frequently used for pain control in cases where conventional medical treatments are ineffective [7]. Research indicates that the chronic administration of dietary components like polyphenols and carotenoids can attenuate inflammation-induced mechanical hyperalgesia by suppressing the hyperexcitability of SpVc wide-dynamic range (WDR) neurons. This is achieved through both peripheral and central cyclooxygenase-2 (COX-2) signaling pathways [8,9]. Among these compounds, myricetin (MYR) stands out for its diverse beneficial properties, including antioxidant, anti-inflammatory, and anticancer effects [10,11,12].

Our study yielded the following significant observations: (i) Chronic administration of CEL or 1/2 CEL + 1/2 AST reversed the mechanical threshold reduction in inflamed rats to control levels by Day 2. (ii) Both CEL and 1/2 CEL + 1/2 MYR administration significantly decreased the mean discharge frequency of SpVc WDR neurons in inflamed rats evoked by non-noxious and noxious mechanical stimuli. (iii) Treatment with CEL or a combination of half-dose CEL + half-dose AST significantly reduced the increased mean spontaneous discharge of SpVc WDR neurons in inflamed rats. Both CEL and the combined treatment also restored the enlarged mean receptive field size to control levels. Importantly, the magnitude of the myricetin (MYR)-mediated inhibition of SpVc neuronal hyperexcitability, a hallmark of hyperalgesia, was nearly commensurate with that of CEL, a selective COX-2 blocker (10 mg/kg, i.p.). This striking similarity indicates that MYR’s therapeutic potential is comparable to CEL, suggesting its promise as a candidate agent for CAM strategies to combat trigeminal inflammatory mechanical hyperalgesia, particularly in conditions like temporomandibular joint disorders and postoperative pain [4,35]. This assertion is further substantiated by the observation that MYR’s inhibitory effect was almost equivalent to that achieved with 1/2 CEL + 1/2 MYR co-administration. A similar method was used in our previous report, which suggested that flavonoids, including naringenin, may have applications in complementary and alternative medicine (e.g., non-specific COX-2 inhibitor, diclofenac) [9]. However, to conclusively establish that myricetin can be a substitute for NSAIDs, further analysis using methods such as isobolographic analysis or the bliss independence model will likely be necessary.

In clinical settings, it is widely recognized that most patients undergoing orthodontic therapy report experiencing pain, including referred pain, for which NSAIDs are commonly prescribed [42]. Orthodontic appliances apply mechanical forces to induce tooth movement, a process often associated with gingival inflammation and bone resorption on the tension side [42]. Given the significant role of prostaglandin E2 (PGE2) in osteoclast-mediated bone remodeling, NSAIDs are known to have detrimental effects on orthodontic patients, such as decreased tooth movement [43,44]. More recently, prolonged intake of the phytochemical resveratrol has been reported to lessen mechanical, ectopic hyperalgesia induced by experimental tooth movement, a condition associated with hyperexcitable SpVc WDR neurons in anesthetized rats [45]. The results suggest that dietary components could offer a promising therapeutic approach for managing ectopic pain, such as the pain associated with orthodontic treatment [45]. In the present study, we found that systemic administration of MYR at a half-dose combination with CEL (1/2 CEL + 1/2 MYR) effectively replaced the half-dose of CEL. This suggests that MYR administration may also attenuate orthodontic treatment-induced ectopic hyperalgesia without the side effects associated with NSAIDs. However, further studies are necessary to fully elucidate this possibility.

**Figure 6:**
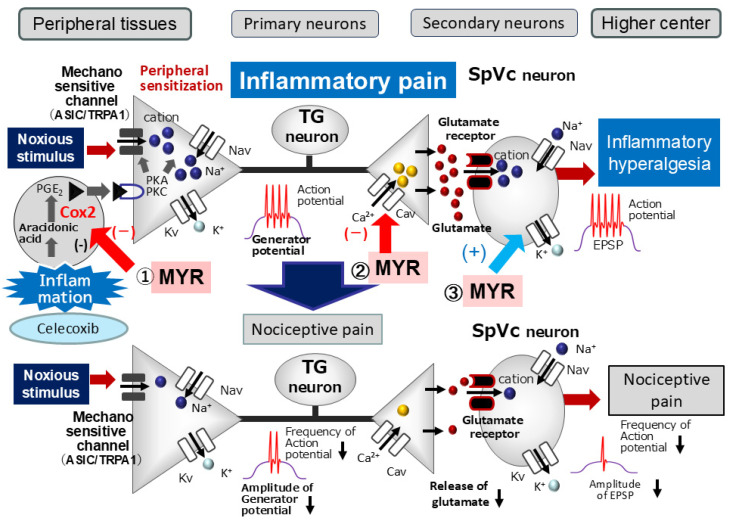
Proposed mechanisms underlying myricetin (MYR)-mediated suppression of inflammation-induced mechanical hyperalgesia. This diagram illustrates the progression of peripheral sensitization and the putative inhibitory role of MYR. Peripheral Sensitization (Inflammation): Following peripheral inflammation, prostaglandin E2 (PGE2) binds to G protein-coupled prostanoid EP receptors on nociceptive peripheral terminals. This binding activates protein kinase A (PKA) and protein kinase C (PKC), which subsequently phosphorylate voltage-gated Na (Nav) and K (Kv) channels and receptors. This phosphorylation lowers the activation threshold of transducer channels, such as transient receptor potential ankyrin 1 (TRPA1), increasing the membrane excitability of the peripheral terminals This results in high-frequency action potentials being sent to the presynaptic central terminals of the spinal trigeminal nucleus caudalis (SpVc). This leads to a significant release of glutamate into the synaptic cleft. This glutamate then binds to postsynaptic receptors, which have been upregulated, thereby increasing excitatory postsynaptic potentials (EPSPs). This barrage of action potentials is then transmitted to higher pain centers, leading to a state of heightened sensitivity known as peripheral sensitization. Myricetin’s (MYR) Inhibitory Mechanism: Systemic administration of MYR is hypothesized to lessen inflammation-induced mechanical hyperalgesia by primarily targeting and suppressing the hyperexcitability of SpVc WDR neurons. This suppressive effect is likely due to a combination of several pathways: Inhibition of peripheral COX-2 signaling—MYR may reduce the inflammatory response at the source by blocking the COX-2 enzyme; Modulation of ion channels—MYR is believed to inhibit central terminal Cav channels while simultaneously activating Kv channels. These combined actions reduce the firing frequency of action potentials in nociceptive nerve terminals. By blocking the transmission of pain signals to the SpVc and higher pain centers, MYR ultimately suppresses hyperalgesia.

This study is the first to directly compare the suppressive potency of MYR with that of CEL, a selective COX-2 antagonist, on inflammation-induced SpVc neuronal excitability. As summarized in Figure 6, our findings indicate that systemic MYR administration likely attenuates inflammation-induced mechanical hyperalgesia primarily by suppressing the hyperexcitability of SpVc WDR neurons. This effect is potentially mediated by a combination of mechanisms, including the inhibition of peripheral COX-2 signaling, modulation of Cav channels, and activation of Kv channels. By acting on these pathways, MYR may inhibit the conduction of pain signals to the SpVc and higher centers, thereby reducing hyperalgesia. Therefore, these results contribute significantly to the development of analgesic drugs with fewer side effects for the treatment and prevention of trigeminal inflammatory pathological pain, including clinical orofacial pain.

## 4. Materials and Methods

The procedures described in this study, approved by the Animal Use and Care Committee of Azabu University (No. 230120-11), fully complied with the ethical guidelines of the International Association for the Study of Pain [46]. Maximum efforts were made to minimize the number of animals used and to alleviate their suffering. Furthermore, all experiments were conducted with the experimenters blinded to the experimental conditions (08:30–12:30).

### 4.1. Inducing Inflammation and Administering Myricetin (MYR) and NSAIDs

Adult male Wistar rats, weighing 215–255 g, were housed under a controlled 12:12 h light–dark cycle (lights on, 07:00–19:00). The acclimatization period of the animals was seven days in this study. To account for documented sex differences in experimental pain responses, and given the as yet undefined underlying mechanisms [34], all experiments were conducted exclusively on male rats. The 20 rats were randomly assigned to one of five experimental groups (n = 4 per group): naive; inflamed; inflamed with myricetin (MYR) treatment (16 mg/kg, i.p.; Sigma-Aldrich, Milano, Italy); inflamed with celecoxib (CEL) treatment (10 mg/kg, i.p.; Sigma-Aldrich, Milano, Italy); and inflamed with co-treatment of CEL (5 mg/kg, i.p.) + MYR (8 mg/kg, i.p.). These doses of MYR and CEL were selected based on previous studies indicating their significant suppression of COX-2 activity in vitro and in vivo studies [18,24]. Each animal was anesthetized with 3% isoflurane to induce inflammation. This was followed by a 0.05 mL injection of Complete Freund’s adjuvant (CFA), a 1:1 oil/saline mixture, into the left facial skin, as previously described [8,9]. Naive rats received an injection of vehicle only (0.9% NaCl) into the left facial skin. For treatment, myricetin (MYR) and celecoxib (CEL) were dissolved in dimethyl sulfoxide (DMSO) and administered once daily for two consecutive days. Daily behavioral experiments were conducted immediately before each administration. The efficacy of MYR in mitigating peripheral inflammation was evaluated by measuring the thickness of CFA-induced edema in the whisker pad region for all groups, a procedure previously outlined [8]. Based on the behavioral analysis for escape threshold, electrophysiological experiments were conducted exclusively on Day 2 in the following groups: naive, CFA-inflamed, CFA-inflamed + MYR, CFA-inflamed + celecoxib (CEL), and MYR 1/2 + CEL 1/2. In this study, every effort was made to minimize the number of animals used and their suffering. Data analysis was performed using multiple comparison tests, with the sample size determined by power analysis to ensure sufficient statistical power to detect significant differences.

### 4.2. Mechanical Escape Threshold

The mechanical threshold for escape behavior was determined as previously described [8,9]. To measure mechanical hyperalgesia, the ipsilateral and contralateral facial skin areas were briefly assessed using a set of von Frey hairs (Semmes-Weinstein Monofilaments, North Coast Medical, Morgan Hill, CA, USA). This was performed one to two days after the Complete Freund’s adjuvant (CFA) or vehicle injection. The escape threshold was determined by applying von Frey stimuli to the whisker pad in ascending order. Each stimulus was administered three times per trial series, with a 5 s interval between applications and between each trial. We defined the escape threshold as the minimum intensity at which a rat retracted its head in response to at least one of the three applications.

### 4.3. Extracellular Single-Unit Recording of SpVc WDR Neuronal Activity

Two days post-injection of CFA or vehicle, electrophysiological recordings were performed as previously outlined [8,9]. Data were collected from 20 adult male Wistar rats. Each animal was initially anesthetized with 3–5% isoflurane and maintained with a continuous anesthetic regimen of medetomidine (0.3 mg/kg), midazolam (4.0 mg/kg), and butorphanol (5.0 mg/kg). Anesthesia depth was confirmed by the absence of a paw pinch response. Supplemental doses of the anesthetic mixture (0.25–0.45 mL/kg/h) were administered intravenously via a jugular vein cannula. Rectal temperature was maintained at 37.0 °C ± 0.5 °C using a homeothermic blanket (Temperature Controller 40-90-8D; FHC Aspen, Tokyo, Japan). All wound margins were continuously infiltrated with a 2% lidocaine solution (Xylocaine) during the experiments. Animals were then positioned in a stereotaxic device (SR-50; Narishige, Tokyo, Japan). A midline incision was made through the neck muscles to expose the medullary brainstem by incising the atlanto-occipital ligament and dura mater. To record extracellular single-unit activity from the spinal trigeminal nucleus caudalis (SpVc), a tungsten microelectrode (3–5 MΩ) was inserted ipsilaterally into the medulla. The microelectrode was precisely positioned using a micromanipulator (SM-11 and MO-10; Narishige, Tokyo, Japan) in 10 µm steps, with guidance from the rat brain atlas of Paxinos and Watson [47]. Neural activity was amplified (DAM80; World Precision Instruments, Sarasota, FL, USA), filtered (0.3–10 kHz), and monitored with an oscilloscope (SS-7672; Iwatsu, Tokyo, Japan). Data were then recorded for subsequent analysis using Power Lab equipment and Chart ver. 5 software (AD Instruments, Oxford, UK), as previously described [8,9].

### 4.4. Experimental Protocols

Extracellular single-unit responses of SpVc WDR neurons to mechanical stimulation of the whisker pad were examined. To prevent sensitization of peripheral mechanoreceptors, a paintbrush was used to swiftly locate the general receptive field on the left whisker pad. We then searched for single units that responded to a range of von Frey hairs, including both non-noxious (0.2, 0.6, 2, 6, 10 g) and noxious (15, 26, 60 g) mechanical stimuli, applied for 5 s with 5 s intervals [8,9]. WDR neurons were defined by their graded response to both non-noxious and noxious mechanical stimulation within their receptive area. Following the identification of nociceptive SpVc WDR neurons, their mechanical stimulation threshold and receptive field size were determined and documented. Neuronal mechanical receptive areas were delineated by applying von Frey hairs to the facial skin and tracing the areas onto a life-sized drawing of the rat [8,9]. WDR neuronal activity was quantified by subtracting background neuronal activity from stimulus-evoked activity. Spontaneous discharge frequencies were assessed over a 2–5 min period. Previous research indicates that (i) WDR neurons in the SpVc region are significantly involved in mechanical hyperalgesia [4], and (ii) NS neurons may convert to WDR neurons after CFA inflammation [1,3]. Consequently, this study focused exclusively on the effects of MYR on nociceptive SpVc WDR neuronal activity, and NS neurons were not investigated. Peristimulus histograms with 100 ms bins were generated for each stimulus. We compared several properties of SpVc WDR neurons across five groups of animals. Specifically, we analyzed their average spontaneous and mechanically induced discharge frequencies, their receptive field size, and their average mechanical thresholds. The groups were as follows: naive, CFA, CFA-inflamed + MYR, CFA-inflamed + CEL, and a combination treatment group (CFA-inflamed + 1/2 MYR + 1/2 CEL). The single-unit recording locations within the SpVc region were identified from micromanipulator readings, documenting the distance from the obex, medial line, and surface of the medullary dorsal horn, with reference to the rat brain atlas, as described in our previous studies [8,9,47].

### 4.5. Data Analysis

Data are presented as the mean ± standard error of the mean (SEM). Statistical evaluation was performed using one-way repeated-measures analysis of variance (ANOVA), followed by Tukey–Kramer or Dunnett tests for post hoc comparisons. Student’s *t*-test was used for electrophysiological data analysis (Excel Statcel 4). A two-sided *p*-value < 0.05 was considered statistically significant.

## 5. Conclusions

Our current research demonstrates that the systemic administration of MYR attenuates inflammatory mechanical hyperalgesia. This effect is likely due to the inhibition of the COX-2 signaling pathway, which reduces the hyperexcitability of nociceptive SpVc WDR neurons. These results support the potential of MYR as a therapeutic agent for CAM strategies aimed at preventing trigeminal inflammatory mechanical hyperalgesia, offering a promising alternative to NSAIDs.

## Data Availability

All data from this study are included in the main body of the article.

## Abbreviations

The following abbreviations are used in this manuscript:

MYR	Myricetin
NSAID	Non-steroidal anti-inflammatory drug
CEL	Celecoxib
SpVc	Spinal trigeminal nucleus caudalis
WDR	Wide-dynamic range
TG	Trigeminal ganglion
CFA	Complete Freund’s adjuvant
CAM	Complementary alternative medicine
COX-2	Cyclooxygenase-2
PGE2	Prostaglandine E2
MAPK	Mitogen activated protein kinase
PKA	Protein kinase A
PKC	Protein kinase C
NMDA	N-methyl-D-aspartate
NR2B	NMDA receptor subtype 2B
Nav	Voltage-gated Na channels
Kv	Voltage-gated K channels
Cav	Voltage-gated Ca channels
TRPA1	Transient receptor protein ankyrin 1
ASIC3	Acid sensing ion channels 3
EPSP	Excitatory postsynaptic potential
EP	Prostanoid E
